# Biliary Calculi

**Published:** 1884-03-20

**Authors:** C. S. Webb

**Affiliations:** Virginia


### BILIARY CALCULI.

BY C. S. WEBB, M. D., OF VIRGINIA.

A few weeks ago I was called out of church to see Mrs. Blank, in an attack of "stomach cramp," to which she has been subject for several years, during which time I have been her family physician. I had never seen her with an attack on, and had been puzzled about the diagnosis; but this time I had no difficulty in deciding that she was passing a gall-stone.

(1) The location of the pain.

(2) It was cutting, tearing.

(3) The countenance of the patient indicative of the keenest agony.

(4) The utter failure of the most powerful anodynes, given in full doses.

(5) The nausea and vomiting.

(6) The sudden subsidence of the pain, and the subsequent soreness.

(7) The presence of bile in the urine the next morning (the attack having lasted all day Sunday and part of the night).

(8) The jaundice hue that was present the next morning, but which faded after twenty-four hours.

The diagnosis was plain, and the question before me was how to empty the gall-bladder of the remaining stones and give this poor sufferer relief. I went at once to my books, "hallowed companions, tutors, ministers." The first thing I did was to study the chemistry of cholesterin, then read several works on practice, after which I went through huge piles of journals, reading every article on the subject. The treatment decided on was as follows:

I began on Thursday, and gave the following:

| | | |
|---|---|---|
| ℞ | Chloroform ............................ | aa. ʒ ij, |
| | Ol. olivæ. ............................ | |
| | Pulv. acaciæ.. ............................ | |
| | Sacch. alb ............................ | |
| | Aq. menth. pip ............................ | ℥ i, |
| | Aquæ, q. s, ............................ | ℥ iv. |

M. Sig. A teaspoonful every four hours. Shake well.

This she took regularly until Saturday morning, when I proceeded to her house armed with a pint of the best olive oil. Having satisfied myself that the stomach was empty, I gave her nearly a gobletful of the olive oil. After forty minutes had elapsed she was placed on the left side, with the hips elevated above the shoulders. In this position she lay for one hour and a half, after which she was permitted to change her position to suit her own comfort.

Three and a half hours after taking the oil she had a copious discharge from the bowels. The fæces were diluted and strained through a wire sieve, when I had the pleasure of collecting about one hundred gall-stones, the largest being about the size of a gardenpea. She has not had the cramp since, and, I trust, will never have it again. She is now taking Stewart's hydrated succinate of the peroxide of iron, a teaspoonful three times a day, as advised by Dr. T. H. Buckler, to prevent, if possible, the crystallization of the cholesterin in future. Now, to what does she owe her delivery ? To the chloroform or to the olive oil, or shall they divide the honors ? The former was given in accordance with the views of Dr. T. H. Buckler, and the latter was given after the manner of Dr. Babbitt, as detailed in the Medical Brief, July, 1881. Bartholow, in his latest work on Practice, says : "It has been gravely proposed to administer chloroform as a solvent of gall-stones." He also says that Dr. Buckler's idea of giving the hydrated succinate of the peroxide of iron "is based on some theoretical notions." Bartholow himself, however, strongly recommends the use of phosphate of soda. May not this, also, be a "theoretical notion ?" He says nothing about olive oil, although much has been written about it in the journals.

Let us not be too tenacious of our own theories and reject those of other physicians, which *may* be better than ours except that *we* did not originate them. Above all, let us not emulate the example of the Frenchman who, when told that the facts were opposed to his theory, shrugged his shoulders and said, "So much the worse for the facts." He intended to keep his theory and let the facts take care of themselves; but a fact will ever remain a fact, despite all our theories to the contrary.

I would humbly suggest that those who write books should read a large number of medical journals and consider carefully the testimony in favor of a certain line of treatment, even though it should be against the author's own practice. And having said this much, I leave the reader to decide what treatment he will adopt for Biliary Calculi.

---

**To Disguise the Taste of Medicines.**—Bitter and nauseous salines are best taken simply diluted with iced water. A mouthful or two of iced water, before and after the dose, to blunt the sense of taste, the dose taken between them in a wineglassful of iced water, renders such medication easy to most persons.—*Squibb's Pharmacist.*

